# The identification of molecular target of (20S) ginsenoside Rh2 for its anti-cancer activity

**DOI:** 10.1038/s41598-017-12572-4

**Published:** 2017-09-29

**Authors:** Yu-Shi Wang, Yingjia Lin, He Li, Yang Li, Zhiguang Song, Ying-Hua Jin

**Affiliations:** 1 Key Laboratory for Molecular Enzymology and Engineering, the Ministry of Education, College of Life Science, Jilin University, Changchun, Jilin 130012 China; 2 College of Chemistry, Jilin University, Changchun, 130012 Jilin China

## Abstract

The 20S ginsenoside Rh2 (G-Rh2) effectively inhibits cancer cell growth and survival in both animal models and cell lines. However, its molecular targets and mechanism of action remain largely unknown. By screening for molecules that interact with (20S)G-Rh2 in a phage display assay, we have identified Annexin A2 as a potential target that mediates its anti-cancer activity. Isothermal titration calorimetry and a cellular thermal shift assay demonstrated that (20S)G-Rh2 directly bound to either recombinant or intracellular Annexin A2. This binding inhibited the interaction between Annexin A2 and the NF-кB p50 subunit, which attenuated the nuclear translocations of NF-кB p50 subunit and reduced the transactivation activity of NF-кB. Correspond to this result, (20S)G-Rh2 treatment significantly down-regulated the expression of IAPs (inhibitors of apoptosis), the well-established NF-кB targets that promote cell survival. Moreover, (20S)G-Rh2 synergized with Annexin A2 inactivation to promote apoptosis. Taken together, this study for the first time suggests a cellular target and a molecular pathway by which (20S)G-Rh2 inhibits cancer cell growth. As over-expression of Annexin A2 was evident in human hepatoma, (20S)G-Rh2 might be a promising natural compound for targeted liver cancer therapy.

## Introduction

Ginseng has been a famous medicinal herb in eastern Asia for over a thousand years, due to its extraordinary efficacy on nourishment, restoration, and disease prevention. Ginsenosides comprise the major effective ingredients of ginseng, presenting various effects on intelligence development, immune response, metabolism promotion, and cancer prevention and treatment^1,2^. Among them, ginsenoside Rh2 (G-Rh2), with a dammarane skeleton (20S), has been shown to induce apoptosis in various cancer cell lines by activating either mitochondrial- or membrane death receptor- mediated apoptosis pathway^3–8^. Moreover, both *in-vivo* and *in-vitro* studies have demonstrated that (20S)G-Rh2 inhibits tumor cell growth and metastasis. Thus, due to its effective anti-cancer activity, (20S)G-Rh2 is considered a promising chemical for cancer therapy^5,7–10^. As (20S)G-Rh2 activates p53 pathway and inhibits NF-кB activity^10,11^, it is reasonable to assume that (20S)G-Rh2 acts as a tumor suppressor via multiple cellular targets and complex signal transduction pathways. However, the cellular targets of (20S)G-Rh2 and the initiating events triggered by this compound remain to be identified.

Annexin A2 is a member of the annexin family. It is a well-known component of the Annexin A2-S100A10 complex, which promotes plasmin generation in vascular endothelial cells and in metastatic cancer cells^12,13^. Full-length Annexin A2 contains binding sites for DNA, mRNA, other proteins, phospholipid, and calcium. These sites provide pleiotropic properties, which allow this protein to participate in multiple signal transduction pathways that are involved in membrane fusion, cell adhesion, DNA synthesis, cell proliferation, and fibrinolysis^14,15^. Importantly, Annexin A2 is over-expressed in various types of tumors, including breast, liver, prostate, and pancreatic tumors. Inactivating of Annexin A2 inhibits cancer cell proliferation and metastasis and sensitizes cancer cells to anti-cancer drugs^16–19^. A recent research showed that the Annexin A2-S100A11 complex facilitates membrane repair in cancer cells and promotes survival of invasive cancer cells^20^. Moreover, intracellular Annexin A2 promotes autophagy and NF-кB activation, which suggested that multi-drug resistance might arise from the over-expression of Annexin A2 in cancer cells^16,19–22^. Thus, Annexin A2 might be a promising molecular target for cancer therapy.

NF-кB is an important transcription factor involved in multiple biological processes, including the immune response, stress response, apoptosis, cell proliferation, and cell metastasis^23^. Abnormal activation of the NF-кB pathway was closely associated with the initiation, promotion, and progression of human cancers^24–27^. NF-кB regulates the expression of various anti-apoptosis genes, including the inhibitor of apoptosis proteins (IAPs), anti-apoptosis members of the Bcl-2 superfamily, and other pro-survival genes, and these regulations promote drug resistance in pancreatic cancer, lung cancer, melanoma, gastric cancer, and hepatocellular carcinoma^16,28–30^. Interestingly, some ginsenosides, like G-Rh2, G-Rg3, and compound K (CK), suppress NF-кB activity^11^. It is tempting to assume that the pro-apoptotic activity of ginsenosides may arise from NF-кB suppression.

In this study, we immobilized (20S)G-Rh2 onto PEGA (polyethylene glycol adipate) resin and performed a phage display to screen for cellular targets of (20S)G-Rh2. We identified 46 potential target genes including Annexin A2. We employed isothermal titration calorimetry and competitive G-Rh2-pulldown assays to assess the interaction between (20S)G-Rh2 and Annexin A2. Here, we demonstrated for the first time that (20S)G-Rh2 directly binds to Annexin A2, which interfered the interaction between Annexin A2 and NF-кB p50 subunit, and thus, down-regulated NF-кB activation and anti-apoptosis gene expression, finally promoted apoptosis in cancer cells.

## Results

### Primary screening of cellular targets of (20S)G-Rh2 by phage display

Five rounds of bio-panning were performed with the (20S/R)G-Rh2-PEGA resin and the T7 Select Human Liver Tumor cDNA phage library. In the fifth round, with an input of 1 × 10^11^ pfu, the elution rates reached 6 × 10^−4^% and 7.3 × 10^−4^% with the (20S)G-Rh2-PEGA and (20R)G-Rh2-PEGA resins, respectively (Tables 1 and S1). Sequences of 181 phage plaques collected by the (20S)G-Rh2-PEGA resin were amplified via PCR followed by gene sequencing. Among these, 95 sequences fell within protein coding regions, 47 sequences fell within non-coding regions, and the other 39 sequences did not belong to the human transcriptome. After filtering out the repeated hits, we finally identified 46 potential targets for (20S)G-Rh2 (Table S2). Fortunately, Annexin A2, a multifunctional tumor-associated protein, was identified with (20S)G-Rh2-PEGA, but not (20R)G-Rh2-PEGA.

**Table 1:** Phage titers obtained after five rounds of bio-panning with (20S)G-Rh2-PEGA resin and the T7 Select Human Liver Tumor cDNA phage library.

Round	Input phage	Elution phage	Elution (%)
Round 1	1 × 10^11^	2 × 10^4^	2 × 10^−5^
Round 2	1 × 10^11^	5.6 × 10^4^	5.6 × 10^−5^
Round 3	1 × 10^11^	3.3 × 10^5^	3.3 × 10^−4^
Round 4	1 × 10^11^	5.5 × 10^5^	5.5 × 10^−4^
Round 5	1 × 10^11^	6 × 10^5^	6 × 10^−4^

### (20S)G-Rh2 bound directly to either recombinant or intracellular Annexin A2

**Figure 1:**
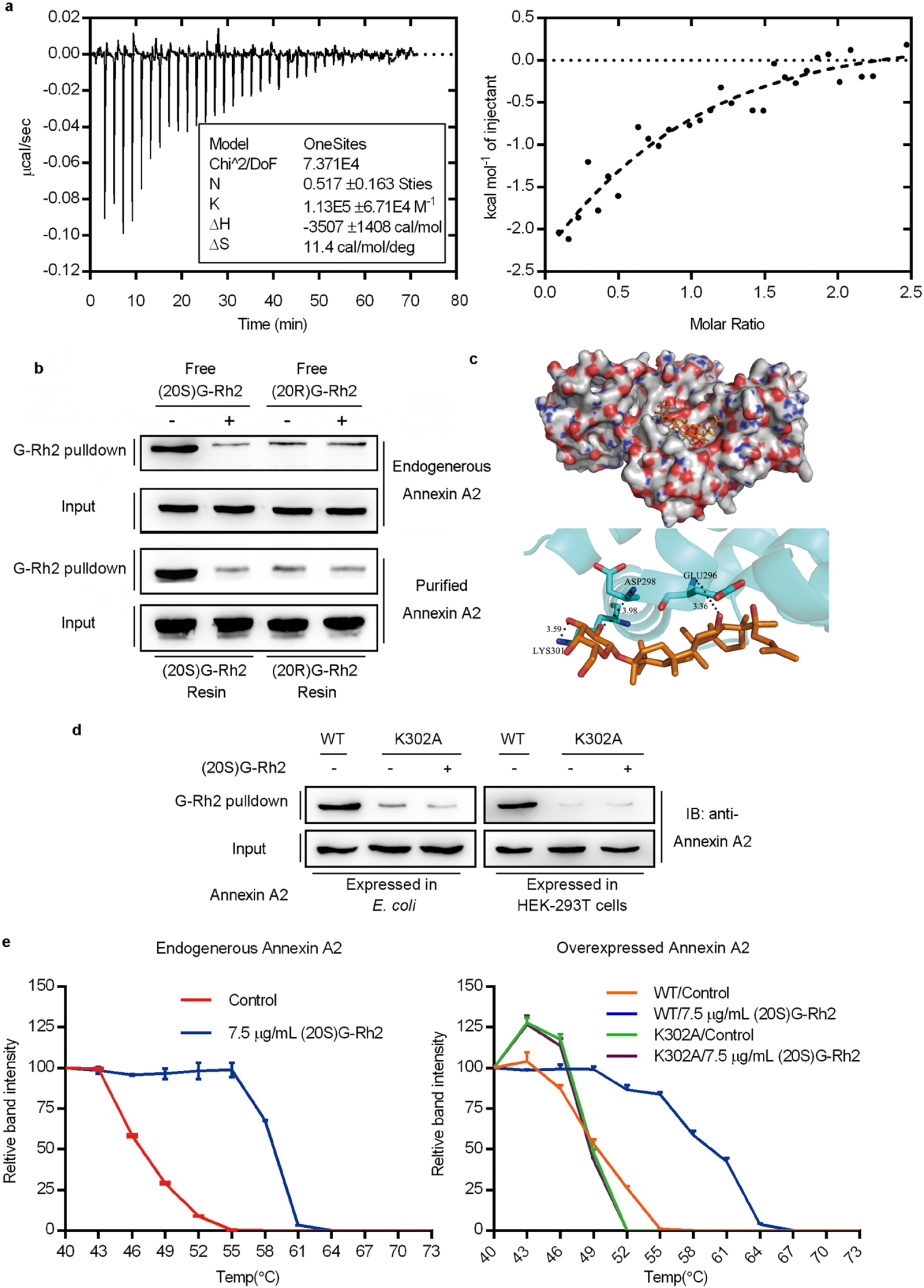
(20S)G-Rh2 directly bound to Annexin A2. (**a**) Results from the isothermal titration calorimetry show binding energy (μcal/s) measured over time (min). (*Inset*) The one-binding-site model results show the kinetics of the interaction. (*Left*) The energy measured (kcal) per mole of injectant indicates the molar ratio of the binding reaction. (**b**) Immunoblot shows the results from competitive Rh2-pulldown assays, performed with the whole-cell lysate (*top*) and purified Annexin A2 (*bottom*). (**c**) Molecular docking illustrations show (*top*) the potential binding site on Annexin A2 and the surrounding amino acids. and (*bottom*) the predicted binding interactions between Annexin A2 (blue) and (20S)G-Rh2 (brown). (**d**) Immunoblot (IB) results of the competitive Rh2-pulldown assays, performed with both wild-type Annexin A2 (WT) and the Annexin A2 K302A mutant. the Annexin A2 proteins were expressed in *E*. *coli* competent cells or HEK-293T cells. (**e**) Cellular thermal shifting assay results show changes in relative band intensities at different temperatures for (*left*) Annexin A2 expressed in HepG2 cells (endogenous) or (*right*) for Annexin A2 wild-type (WT) and K302A mutant constructs expressed in HEK-293T cells (over-expressed). All data are shown as the mean ± SD and the experimental points show the average of at least triplicates. All experiments were repeated at least 3 times.

To confirm the interaction between (20S)G-Rh2 and intact Annexin A2, we performed isothermal titration calorimetry with a purified recombinant full-length Annexin A2. The result showed that (20S)G-Rh2 bound to Annexin A2 in an equimolar ratio, with an association constant of 1.13 × 10^−5^ M^−1^ and a binding free energy of −3.507 kJ/mol (Fig. 1a). A competitive pull-down assay showed that both the endogenous Annexin A2 expressed in HepG2 cells and the purified recombinant Annexin A2 interacted with the (20S)G-Rh2-PEGA resin, and this interaction was remarkably reduced by free (20S)G-Rh2. In contrast, little binding was observed with the (20R)G-Rh2-PEGA resin, and no change was observed after adding free (20R)G-Rh2 (Fig. 1b).

The nucleotide sequence of Annexin A2 obtained from the phage display encoded amino acids 143 to 308 within the Annexin A2 protein, covering the entire domain III and parts of domains II and IV (Figure S1a). Next, we performed a molecular docking analysis, based on the published crystal structure of Annexin A2 (PDB ID: 2HYU). The optimum binding site for (20S)G-Rh2 was located within domain IV of Annexin A2 and three amino acid residues were involved in the interaction with (20S)G-Rh2: the ε-amino group of Lys302 and the amino groups of Asp299 and Glu297 (Fig. 1c). A competitive pull-down assay with the K302A mutant of Annexin A2 (Annexin A2-K302A) showed that the mutant did not interact with (20S)G-Rh2-PEGA resin, either when Annexin A2-K302A was over-expressed in HEK-293T cells or when it was expressed and purified from prokaryotic cells (Fig. 1d). These data suggest that (20S)G-Rh2 directly and specifically bound to Annexin A2 and Lys302 was a critical amino acid residue for this binding.

To examine whether (20S)G-Rh2 can interact with intracellular Annexin A2, we performed cellular thermal shift assays in HepG2 cells. The temperature that elicited a half-maximal thermal response (T_m_50) for Annexin A2 shifted from 47.5 °C to 57.2 °C, after adding (20S)G-Rh2 (Fig. 1e, Table S4). Next, we tested Annexin A2 obtained by transfecting a vector that carried c-myc fused to either the wild-type Annexin A2 (Annexin A2-WT) or K302A mutant of Annexin A2 (Annexin A2-K302A) in HEK-293T cells. (20S)G-Rh2 caused the T_m_50 of Annexin A2-WT to shift from 49.48 °C to 58.44 °C, but the T_m_50 of the Annexin A2-K302A remained stable at around 48 °C (Figs 1e and S1b). Taken together, these data demonstrated that Annexin A2 was a target of (20S)G-Rh2.

### (20S)G-Rh2 inhibited NF-кB activation and down-regulated anti-apoptosis gene expression

**Figure 2:**
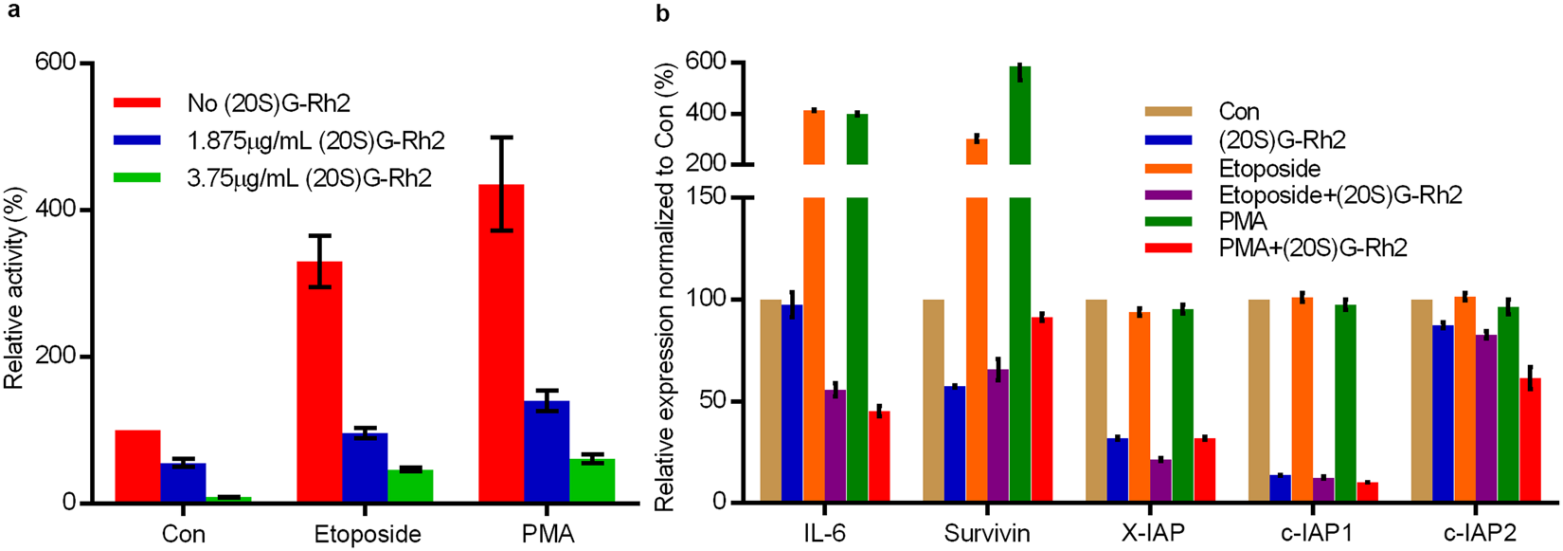
(20S)G-Rh2 inhibits NF-кB transcription activity and down-regulates anti-apoptosis gene expression. (**a**) Luciferase reporter gene assay results show how (20S)G-Rh2 or (20R)G-Rh2 affect NF-кB transcriptional activity in the absence (Con) or presence of 100 ng/mL PMA or 25 μg/mL etoposide. (**b**) Semi-quantitative PCR results show how (20S)G-Rh2 affects the mRNA levels of IL-2, X-IAP, HIF1-α, Survivin, IL-6, c-IAP1, c-IAP2, and Bcl-xL, in the absence (−) or presence (+) of 100 ng/mL PMA or 25 μg/mL etoposide. All data are shown as the mean ± SD and the experimental points show the average of at least triplicates. All experiments were repeated at least 3 times.

Previous study has shown that Annexin A2 up-regulates the transcription activity of NF-κB by binding to and facilitating nuclear translocation of the p50 subunit of NF-кB^16^. Moreover, the anti-apoptosis genes, including c-IAP1, c-IPA2, X-IAP, Survivin, and Bcl-xL were regulated by NF-κB^31^, leading us to speculate that the binding of (20S)G-Rh2 to Annexin A2 might modulate Annexin A2-mediated NF-кB activation and expression of these anti-apoptosis proteins. To examine this possibility, we performed a luciferase reporter gene assay showing that (20S)G-Rh2 (1.875 μg/mL and 3.75 μg/mL) effectively inhibited NF-кB activation in a dose dependent manner, both in the resting state and during NF-кB activation stimulated by 100 ng/mL PMA or 25 μg/mL etoposide (Fig. 2a) in HepG2 cells. Expression of anti-apoptosis genes, including c-IAP1, cIPA2, X-IAP, and Survivin, in addition to the NF-кB target gene, IL-6 (Figs 2b and S2a,b) was also markedly down-regulated with (20S)G-Rh2 treatment, indicating that (20S)G-Rh2 can inhibit NF-кB transcription activity in HepG2 cells.

### (20S)G-Rh2 inhibited Annexin A2 binding to NF-кB p50 subunit

**Figure 3:**
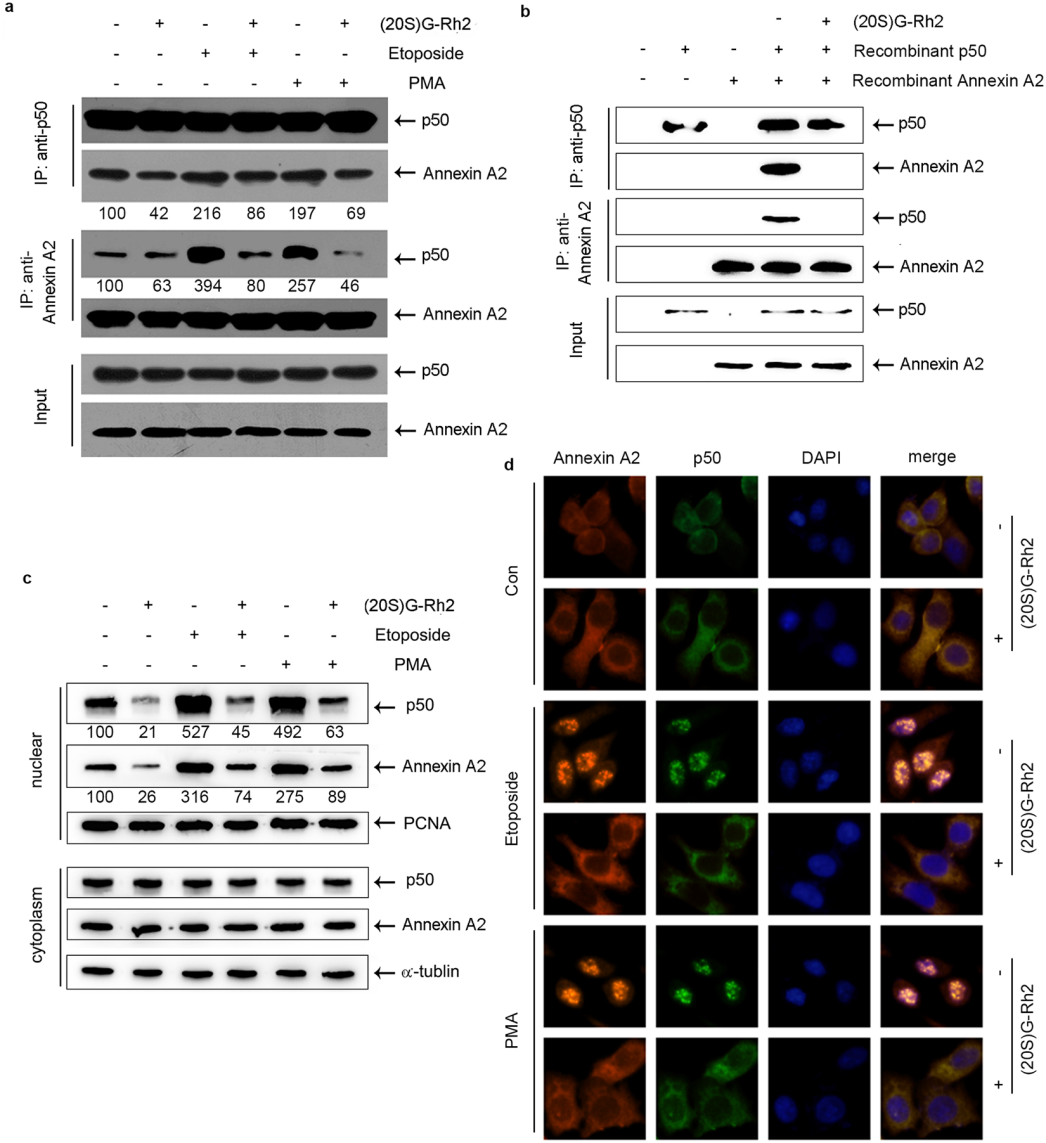
(20S)G-Rh2 inhibits the interaction between Annexin A2 and NF-кB p50 subunit. (**a**) Immunoblot shows immunoprecipitated proteins from HepG2 cells and treated without (−) or with (+) 3.75 μg/mL (20S)G-Rh2, 100 ng/mL PMA, or 25 μg/mL etoposide. Immunoprecipitation (IP) of the whole-cell lysate is with the anti-NF-кB p50 subunit antibody (*top two rows*) and the anti-Annexin A2 antibody (*middle two rows*). (**b**) *E*. *coli*. cells are transformed with non-tagged Annexin A2 and NF-кB p50 subunit. Cell lysates are mixed and treated without (−) or with (+) 3.75 μg/mL (20S)G-Rh2. Cell lysates are immunoprecipitated with anti-NF-кB p50 subunit antibody (*top two rows*) or anti-Annexin A2 antibody (*middle two rows*). (**c**) Immunofluorescence images show the distribution of Annexin A2 (red) and NF-кB (green) in HepG2 cells treated without (−) or with (+) 3.75 μg/mL (20S)G-Rh2, and also treated without (Con) or with either 100 ng/mL PMA or 25 μg/mL etoposide for 2 h. (**b**) Immunoblot of separated nuclear and cytoplasmic proteins shows the distributions of Annexin A2 and NF-кB under the indicated conditions.

To test whether the inhibition of NF-кB activity by (20S)G-Rh2 in HepG2 cells was resulted from the attenuated interaction between Annexin A2 and NF-кB p50 subunit, we performed an immunoprecipitation after cells were treated with 3.75 μg/mL (20S)G-Rh2 without or with NF-кB activators, PMA (100 ng/mL) or etoposide (25 μg/mL). The interaction between Annexin A2 and NF-кB p50 significantly inhibited with (20S)G-Rh2 treatment, both in resting cells and NF-кB activator treated cells, in which the interaction was largely enhanced (Fig. 3a). To rule out the possibility that other mammalian proteins may mediate the inhibitory effect of (20S)G-Rh2 on the interaction between Annexin A2 and NF-кB p50 subunit, we expressed Annexin A2 and NF-кB p50 subunit in *E*. *coli* BL21, and performed immunoprecipitation with these cell lysates. Prokaryotic cell-expressed Annexin A2 and NF-кB p50 subunit bound to each other, and their interaction was also inhibited largely by (20S)G-Rh2 (Fig. 3b).

Next, we examined whether the nuclear localization of the Annexin A2 and NF-кB p50 subunit was inhibited by (20S)G-Rh2 treatment. Either immune-blot or immunofluorescence clearly showed that the nuclear localization of Annexin A2 and NF-кB p50 subunit were significantly inhibited in the resting cells and in NF-кB activator stimulated cells with (20S)G-Rh2 treatment (Fig. 3c and d). Taken together, we suggest that binding of (20S)G-Rh2 to Annexin A2 effectively inhibited the interaction between Annexin A2 and NF-кB p50 subunit, and their nuclear localization.

### Annexin A2-knockdown enhanced (20S)G-Rh2-induced apoptosis

**Figure 4:**
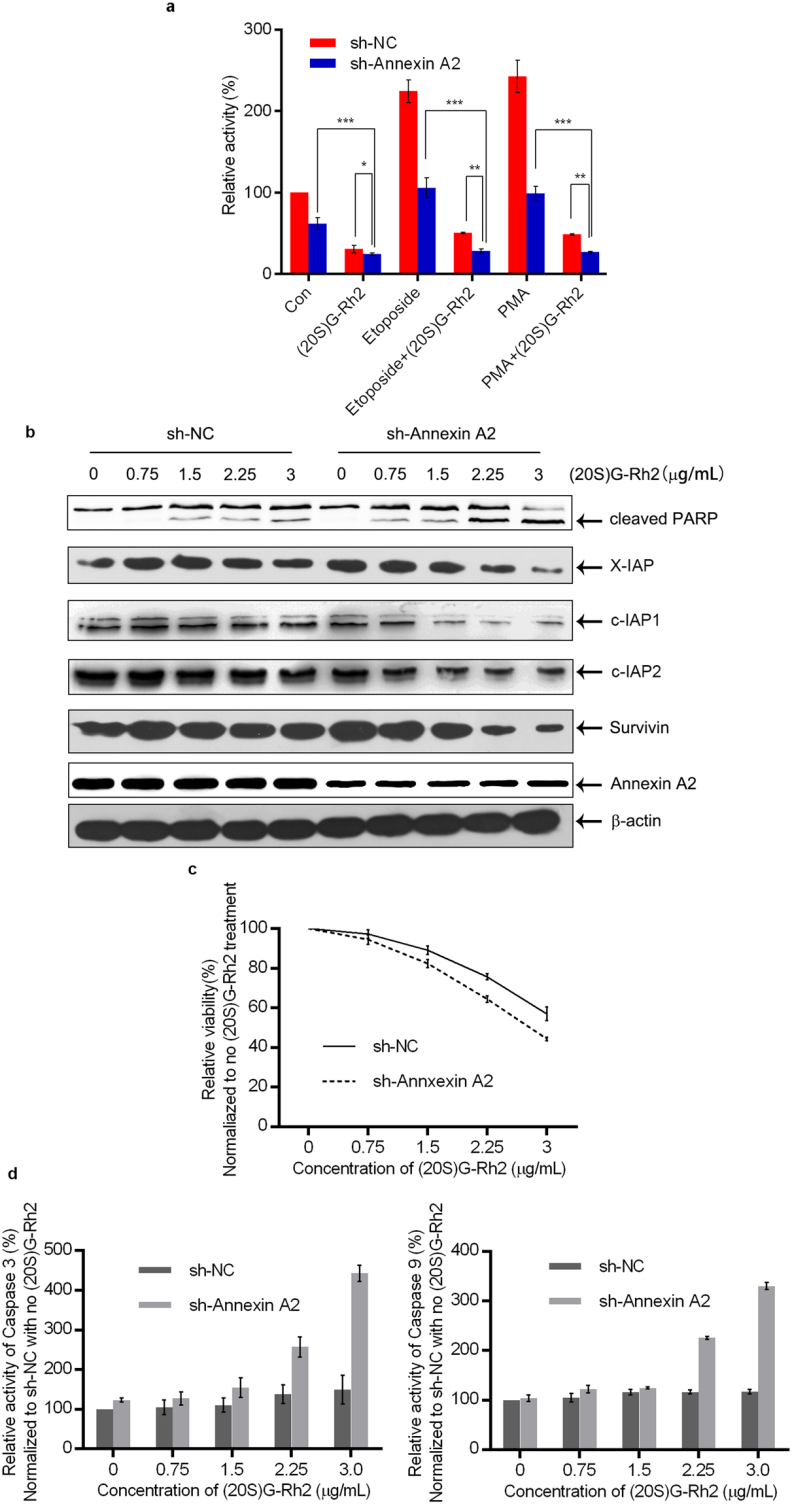
(20S)G-Rh2 inhibits Annexin A2-mediated NF-кB activation of anti-survival signal transduction. (**a**) Luciferase reporter assay results show NF-кB activation in the presence (sh-NC) or absence (sh-Annexin A2) of Annexin A2 expression, in the absence (con) or presence of 3.75 μg/mL (20S)G-Rh2 and with 100 ng/mL PMA or 25 μg/mL etoposide. (**b**) Immunoblots show protein levels of X-IAP, c-IAP1, c-IAP2, and Survivin, and PARP cleavage in HepG2 cells and Annexin A2-knockdown HepG2 cells exposed to the indicated concentrations of (20S)G-Rh2. (**c**) Cell viability in control (sh-NC) and Annexin A2-knockdown (sh-Annexin A2) HepG2 cells exposed to the indicated concentrations of (20S)G-Rh2 for 48 h. (**d**) Relative activity of caspase 3, and 9 in HepG2 cells with normal Annexin A2 levels (sh-NC) or low Annexin A2 levels (sh-Annexin A2) exposed to low concentrations of (20S)-G-Rh2. All data are shown as the mean ± SD *P < 0.05, **P < 0.01, and ***P < 0.001 and the experimental points show the average of at least triplicates. All experiments were repeated at least 3 times. Statistical analyses were performed using Student’s t-test.

Next we examined whether the inactivation of Annexin A2 by gene knockdown reduced NF-кB activity in HepG2 cells, as previously observed in pancreatic cancer cells^16^. The report gene assay showed that NF-кB activity significantly down-regulated in Annexin A2-knockdown HepG2 cells compared to control cells, under both resting state and treatment of NF-кB activator (Fig. 4a). The expression of NF-кB-regulated genes including c-IAP1, c-IAP2, X-IAP, Survivin, IL-2, and IL-6 were also obviously down-regulated in Annexin A2-knockdown cells (Figure S3a). Because, (20S)G-Rh2 effectively inhibited the formation of Annexin A2-p50 complex and NF-кB activation, we asked whether (20S)G-Rh2 synergized with the Annexin A2 knockdown to inhibit NF-кB activation and the expression of downstream genes. As expected, the inhibitory effect of (20S)G-Rh2 on NF-кB activity was dramatically enhanced in Annexin A2-knockdown cells compared to that of control cells (Fig. 4a). Similarly, (20S)G-Rh2-induced down-regulation of c-IAP1, c-IAP2, X-IAP, Survivin, and IL-6 was much more significant in Annexin A2-knockdown cells than that of control cells (Fig. 4b). Next we compared cell viability of Annexin A2-knockdown cells to that of control cells upon (20S)G-Rh2 treatment. The results showed that the survival rate of Annexin A2-knockdown cells was obviously lower than that of control cells with (20S)G-Rh2 treatment (Fig. 4c, Table 2). The activation of both caspase 3 and 9 was much more evident in Annexin A2-knockdown cells than that of control cells upon (20S)G-Rh2 treatment (Figs 4d and S4b). In addition, PARP cleavage occurred at a lower (20S)G-Rh2 concentration in Annexin A2-knockdown cells, compared to control cells (Fig. 4b). Thus, silencing of Annexin A2 rendered cells largely sensitive to (20S)G-Rh2 treatment.

**Table 2:** IC50 of (20S)G-Rh2 in HepG2 cells that expressed Annexin A2 at control (sh-NC) or low levels (sh-Annexin A2).

IC50 of (20S)G-Rh2(μg/mL)	sh-NC	sh-Annexin A2
Con	3.128	2.886
100 ng/mL PMA	2.948	2.222
25 μg/mL etoposide	2.893	2.357

### K302A mutant of Annexin A2 was resistant to (20S)G-Rh2 effect

**Figure 5:**
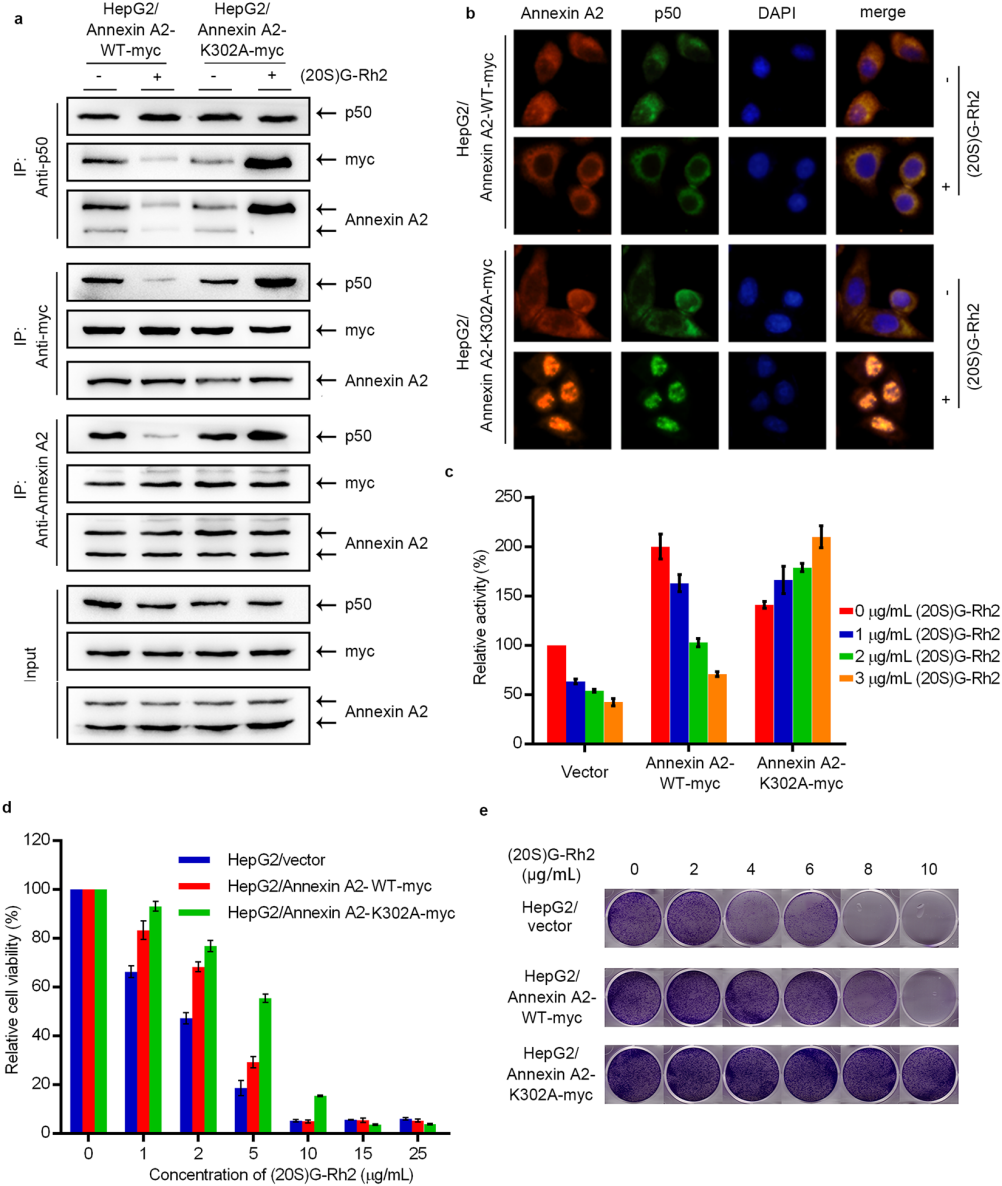
K302A mutant protects cancer cells from the inhibitory effect of NF-кB induced by (20S)G-Rh2. HepG2 cells are transfected with Annexin A2-WT-myc and Annexin A2-K302A respectively. (**a**) Transfected cells are treated without (−) or with (+) 3.75 μg/mL (20S)G-Rh2 for 12 h, whole-cell lysate are immunoprecipitated with the anti-NF-кB p50 subunit antibody, anti-myc antibody and anti-Annexin A2 antibody. (**b**) Immunofluorescence images show the distribution of Annexin A2 (red) and NF-кB (green) in transfected HepG2 cells treated without (−) or with (+) 3.75 μg/mL (20S)G-Rh2 for 2 h. (**c**) Luciferase reporter assay results show NF-кB activation of transfected HepG2 cells exposed to the indicated concentrations of (20S)G-Rh2 for 12 h. (**d**) Transfected cells (1 × 10^3^ per well) are plated onto 6-well plate and exposed to the indicated concentrations of (20S)G-Rh2. Then cells are cultured for 1 week followed by crystal violent staining. (**e**) Transfected cell (5 × 10^3^ per well) are plated onto 96-well plate and exposed to the indicated concentrations of (20S)G-Rh2. Then cells are cultured for 72 h followed by MTT assay. All data are shown as the mean ± SD and the experimental points show the average of at least triplicates. All experiments were repeated at least 3 times.

To provide further evidence for the notion that the inhibitory effect of (20S)G-Rh2 on NF-кB activity is mediated by the direct binding of (20S)G-Rh2 to Annexin A2 protein. For this end, we examined whether over-expression of Annexin A2-K302A rendered cells resistant to (20S)G-Rh2 triggered signal transductions, including the interaction between Annexin A2 and NF-кB p50, and their nuclear localization, as well as the subsequent NF-кB activation in HepG2 cells. The interaction between Annexin A2 and NF-кB p50 subunit and nuclear localization of these two proteins were evidently inhibited by (20S)G-Rh2 treatment in wild-type Annexin A2-over-expressed HepG2 cells. By contrast, neither the interaction between Annexin A2-K302A and NF-кB p50 subunit nor their nuclear localization was down-regulated by (20S)G-Rh2 treatment, indicating that the binding of (20S)G-Rh2 to Annexin A2 was critical to inhibit the interaction between Annexin A2 and NF-кB p50 subunit and subsequent their nuclear localization (Fig. 5a,b). The interaction between Annexin A2-K302A and NF-кB p50 subunit was increased to an extent by (20S)G-Rh2 treatment in Annexin A2-K302A transfectants (Fig. 5a). NF-кB activity was inhibited in a dose-dependent manner in wild-type Annexin A2 transfected cells with (20S)G-Rh2 treatment. By contrast, over-expression of Annexin A2-K302A abolished the inhibitory effect of (20S)G-Rh2 on NF-кB activity (Fig. 5c). These data suggest that (20S)G-Rh2 down-regulates NF-кB activity mainly by targeting Annexin A2 in HepG2 cells.

Our next goal was to provide evidence for the notion that Annexin A2 is an important target protein of (20S)G-Rh2 to execute its anti-cancer activity. For this end, we examined whether ectopic over-expression of Annexin A2-K302A rendered HepG2 cells resistant to (20S)G-Rh2-induced cell death more efficiently than over-expression of wild-type Annexin A2. MTT (3-(4,5-dimethylthiazol-2-yl)-2,5-diphenyltetrazolium bromide) assay showed that the IC_50_ of (20S)G-Rh2 increased ~2-fold in wild-type Annexin A2 transfectants, and ~3-fold in Annexin A2-K302A transfectants, respectively, relative to control cells(Fig. 5d, Table 3). Plate clone formation assay also clearly showed that Annexin A2-K302A transfectants prevented (20S)G-Rh2 induced cell death much more significantly than wild-type Annexin A2 transfectants (Fig. 5e), suggesting that the anti-survival activity of (20S)G-Rh2 on cancer cell was primarily executed by targeting Annexin A2 in HepG2 cells.

**Table 3:** IC50 of (20S)G-Rh2 in HepG2 cells that expressed wide type and K302A mutant of Annexin A2.

	IC50 of (20S)G-Rh2(μg/mL)
HepG2/vector	1.648
HepG2/Annexin A2-WT	2.879***
HepG2/Annexin A2-K302A	4.412***

## Discussion

(20S)G-Rh2 has been suggested to inhibit cancer development, progression, and metastasis^7–10^. Our previous study showed that (20S)G-Rh2 triggered the mitochondrial translocation of Bax and Bak, promoted cytochrome *c* release, and initiated caspase 9-dependent apoptosis^4,32^. (20S)G-Rh2 also induced p53 activation, and triggered caspase 8-dependent apoptosis, by up-regulating Fas^10^. A number of studies suggested that (20S)G-Rh2-induced apoptosis was associated with the activation of cyclin A-Cdk2, PKC-delta, and JNK1^4,5,11^. Thus, (20S)G-Rh2 is likely to interact with multiple cellular targets, and it modulates numerous signal transduction pathways. Because, the epimer of (20S)G-Rh2, (20R)G-Rh2, exhibited little cytotoxicity in tumor cells^4,9^, the target specificity of (20S)G-Rh2 should determine its distinct functions. Therefore, exploring the cellular targets of (20S)G-Rh2 is a key approach to understand its efficacy and mechanism of action.

Here, we performed a phage display, a systematic method widely used for the discovery of small molecule–protein interactions. We screened for proteins that showed pivotal interactions with (20S)G-Rh2, using (20R)G-Rh2 as a negative control. Fortunately, we found Annexin A2, a cancer-promoting protein, which could specifically bind to the (20S)G-Rh2-PEGA resin, but not the (20R)G-Rh2-PEGA resin. We demonstrated the interaction between (20S)G-Rh2 and purified Annexin A2 by an isothermal titration calorimetry assay (Fig. 1a). The competitive pulldown assays further confirmed that both endogenous and purified Annexin A2 specifically bound to (20S)G-Rh2 (Fig. 1b), but little interaction was observed between Annexin A2 and (20R)G-Rh2. Molecular docking suggested that the optimum binding site of (20S)G-Rh2 was located in a groove within domain IV of Annexin A2, in addition, lys302 was a key amino acid in the interaction (Figs 1c and S1a). In another competitive pulldown assay with the mutant version of Annexin A2, Annexin A2-K302A, we demonstrated that the mutation at lys302 caused the loss of (20S)G-Rh2 binding (Fig. 1d). In addition, a cellular thermal shift assay showed that (20S)G-Rh2 could bind to Annexin A2 in living cells (Figs 1e and S1b). Those results demonstrated that Annexin A2 was a cellular target of (20S)G-Rh2 and that lys302 was a key amino acid in the engagement between Annexin A2 and (20S)G-Rh2.

Annexin A2 plays multiple roles in tumor cell proliferation and metastasis^16–21^. A previous report has indicated that intracellular Annexin A2 functioned as an anti-apoptosis factor and enhanced drug resistance by interacting with NF-кB p50 subunit^16^. Thus, the interaction between Annexin A2 and (20S)G-Rh2 may be linked functionally to the NF-кB pathway within a cell. As expected, (20S)G-Rh2 inhibited NF-кB activation in human liver cancer HepG2 cells even in the presence of NF-кB activators, either etoposide or PMA (Fig. 2a). A typical target gene of NF-кB, IL-6, and important anti-apoptosis genes, including c-IAP-1, c-IAP-2, X-IAP, and Survivin, which were also regulated by NF-кB were down-regulated by (20S)G-Rh2 (Figs 2b and S2a,b). An immunoprecipitation assay demonstrated that the interaction between Annexin A2 and NF-кB p50 subunit was significantly inhibited by (20S)G-Rh2 (Fig. 3a). Another immunoprecipitation assay with *E*. *coli* cell lysate expressing recombinant Annexin A2 and NF-кB p50 subunit also showed that (20S)G-Rh2 inhibited their interactions, indicating that the inhibitory effect of (20S)G-Rh2 on the formation is executed without any other mammalian protein(Fig. 3b). Further, the nuclear translocation of either Annexin A2 or NF-кB p50 subunit was also significantly inhibited by (20S)G-Rh2 (Fig. 3c,d), which could directly affect the transcriptional activity of NF-кB. To determine whether (20S)G-Rh2 inhibited NF-кB activation in an Annexin A2-dependent manner, we performed gene silencing or over-expression of Annexin A2, followed by (20S)G-Rh2 treatment. The results showed that down-regulation of Annexin A2 restricted NF-кB activation and showed synergistic effects with (20S)G-Rh2 (Figs 4a and S3b). However, the over-expression of Annexin A2-K302A promoted nuclear translocation of NF-кB p50 subunit and prevented (20S)G-Rh2-induced NF-кB inhibition regardless of (20S)G-Rh2 (Fig. 5a,b). Taken together, these results indicated that (20S)G-Rh2 induced NF-кB suppression mainly by targeting Annexin A2.

To study Annexin A2-mediated anti-cancer activity of (20S)G-Rh2, we conducted MTT and plate clone formation assay. Either over-expression of wild-type Annexin A2 or that of Annexin A2-K302A rendered HepG2 cells resistant to cell death induced by (20S)G-Rh2 treatment. Importantly, the resistant effect was more evident in Annexin A2-K302A transfectants than that of wild-type Annexin A2 transfectants (Fig. 5d,e, and Table 3). By contrast, silencing of Annexin A2 rendered cells much more sensitive to (20S)G-Rh2-induced apoptosis (Figs 4b–d and S4d).

Annexin A2 is over-expressed in several cancer cell lines, and it promotes cancer cell survival and metastasis^12^. To check the Annexin A2 expression in human hepatoma, we analyzed published patients’ data via Oncomine (http://www.oncomine.org), a free online bioinformatic resource of cancer transcriptome data. It collects clinical mRNA array data of different genes from different patients all over the world. As shown in Figure S6a, Annexin A2 mRNA level was significantly elevated in hepatocellular carcinoma (P < 0.001) and the ROC curve generated based on the mRNA level of Annexin A2 indicated that Annexin A2 can be a biomarker of high accuracy (AUC > 0.9) in hepatocellular carcinoma (Figure S6b). The inhibition of Annexin A2 by gene silencing or small chemical inhibitors, like Withaferin A, significantly reduced cancer cell growth and tumor metastasis^33^. Thus, Annexin A2 might be an important target protein, and (20S)G-Rh2 is a promising natural compound for anti-hepatoma therapies.

In summary, we demonstrated that (20S)G-Rh2 directly bound to Annexin A2, which inhibited the interaction between Annexin A2 and NF-кB p50 subunit. This interference promoted apoptosis by inhibiting NF-кB activity and down-regulating anti-apoptosis gene expression. This study was the first to identify the cellular target of (20S)G-Rh2, and we revealed how (20S)G-Rh2 promoted apoptosis in cancer cells.

## Materials and Methods

### Cell lines, reagents, and plasmids

HepG2, and HEK-293T cells were obtained from the American Type Culture Collection (ATCC, Rockville, MA, USA). Dulbecco’s Modified Eagle Medium (DMEM) was obtained from Gibco BRL (Grand Island, NE, USA). The following reagents were used: (20S)G-Rh2 (Sigma), (20R)G-Rh2 (Sigma), phorbol myristate acetate (PMA. Sigma), and etoposide (Sigma). The ginsenosides were dissolved in 75% alcohol. PMA and etoposide were dissolved in DMSO. For use in phage display, we purchased the T7 select human liver tumor cDNA phage library and host *Escherichia coli* BLT5615 bacteria cells from Millipore (MA, USA.). We used the following primary antibodies: rabbit anti-PARP (Santa Cruz), rabbit anti-c-IAP1 (Santa Cruz), rabbit anti-NF-кB p50 subunit (Santa Cruz), rabbit anti-PCNA (Santa Cruz), rabbit anti-myc (Santa Cruz), rabbit anti-cleaved caspase 3 (Cell Signaling), rabbit anti-cleaved caspase 9 (Cell Signaling), mouse anti-Annexin A2 (Santa Cruz), mouse anti-Survivin (Santa Cruz), mouse anti-β-actin (Santa Cruz), mouse anti-α-tubulin (Santa Cruz), mouse anti-X-IAP (Santa Cruz), and mouse anti-c-IAP2 (Cell Signaling). We used the following secondary antibodies: HRP-conjugated goat anti-mouse IgG (Pierce), HRP-conjugated goat anti-rabbit IgG (Pierce), Cy™3 affinipure donkey anti-mouse IgG (Jackson ImmunoResearch Inc., PA, USA), and Alexa Fluor® 488 affinipure donkey anti-Rabbit IgG (Jackson ImmunoResearch Inc., PA, USA). We synthesized the genes that encoded human Annexin A2 and human NF-кB p50 subunit with PCR, and we cloned the sequences into the pEXS-CG vector (provided by Professor Fei Sun’s group, at the Institute of Biophysics of Chinese Academy of Sciences). The resulting prokaryotic vectors allowed expression of non-tagged Annexin A2 (pEXS-Annexin A2), non-tagged NF-кB p50 subunit (pEXS-p50), and C-terminal GST-tagged Annexin A2 (pEXS-CG-Annexin A2). To prepare Annexin A2 mutant, we engineered a single point mutation within the pEXS-CG-Annexin A2 construct, and the result was called pEXS-CG-Annexin A2-K302A. We also constructed eukaryotic vectors that carried c-myc fused to the wild-type Annexin A2 (pcs4-Annexin A2-WT-myc) and c-myc fused to the K302A mutant of Annexin A2 (pcs4-Annexin A2-K302A-myc). We used the short hairpin RNA (shRNA) vector, pGPU6-GFP-Neo-Annexin A2, to create shRNA-Annexin A2 gene silencers, which knocked down Annexin A2 expression to different degrees in RNA interference assays (GenePharma, Jiangsu, China). The luciferase reporter gene assays were performed with the following plasmids: pNFκB-TA-luc (Beyotime, Shanghai, China) and pRL-CMV (Promega, WI, USA).

### Synthesis of ginsenoside (20S/R)G-Rh2-PEGA resin

We washed 750 mg of amino PEGA resin (Millipore, MA, USA) three times with pyridine, and added 0.33 mmol of nitrophenyl bromoacetate (Alfa Aesar, MA, USA). The resin was stirred at room temperature for 3 h, filtered, and washed three times with 10 mL each of dichloromethane (CH_2_Cl_2_), MeOH, and N, N-dimethylformamide (DMF). The brominated PEGA resin was mixed with 0.3 mmol (20S)G-Rh2 and 0.3 mmol K_2_CO_3_ in DMF. The mixture was stirred at room temperature for 3 days, filtered, and washed three times with 10 mL MeOH. We prepared (20R)G-Rh2-PEGA resin in the same way. The PEGA resins were balanced with TBST (10 mM Tris-HCl, pH 8.0, 150 mM NaCl, and 0.1% TWEEN-20) before use.

### Phage display screening

We performed phage display screening with the T7 Select Human Liver Tumor cDNA phage library (Millipore, MA, USA). The original library was amplified by infecting host cells with up to 10^11^ phage forming units (pfu)/mL. Then, 1 mL of amplified phage was incubated with 100 μL native PEGA resin overnight at 4 °C to break non-specific bonds. Next, 800 μL of pre-cleared phage was mixed with 200 μL (20S)G-Rh2-PEGA resin or (20R)G-Rh2-PEGA resin, and incubated overnight at 4 °C. The resin was washed three times with 1 mL of TBST for three times and incubated with 200 μL elution buffer (1% SDS) for 30 min at room temperature with gentle shaking. Next, 5 μL of the eluted fraction was inoculated into 5 mL of *E*. *coli* BLT5615 cells and incubated for 3 h at 37 °C. Phage-infected cells were mixed with NaCl to a final concentration of 0.5 M and centrifuged at 800 × *g* for 5 min. The supernatants were utilized for subsequent bio-panning. The number of pfu was determined by performing a phage titer, before the bio-panning step, according to the manufacturer’s protocol.

### Sequence analysis of selected phage recombinants

After the final bio-panning step, phages were diluted to obtain an individual plaque. The plaques were resuspended in 100 μL of lysis buffer (10 mM EDTA, pH 8.0), heated at 65 °C for 10 min, and briefly vortexed. The lysate was cooled down to room temperature and centrifuged at 12,000 × *g* at 4 °C for 3 min. The supernatant served as a PCR template, and amplification was performed according to the manufacturer’s protocol (Primers: 5′-GGAGCTGTCGTATTCCAGTC-3′ and 5′-AACCCCTCAAGACCCGTTTA-3′. thermal cycler program: One cycle at 94 °C for 5 min, followed by 40 cycles at 94 °C for 30 s, then 58 °C for 90 s, and 72 °C for 90 s, and finally, an extension at 72 °C for 10 min). Sequences were aligned to the human transcriptome stored in the NCBI database, which offers multiple gene and peptide resources.

### Molecular docking

We downloaded the three-dimensional structure of (20S)G-Rh2 (PubChem CID: 119307) from the NCBI Pubchem Compound database (http://www.ncbi.nlm.nih.gov/pccompound), and we downloaded the crystal structure of Annexin A2 (PDB ID: 2HYU) from the RCSB Protein Data Bank (http://www.rcsb.org/pdb). We performed molecular docking with AutoDock tools (version 4.2.6) with the default setting, based on the Lamarckian Genetic Algorithm (Scripps Research Institute, La Jolla, CA, USA). We processed the optimum structure of the complex with the Discovery Studio 4.0 Visualizer (BIOVIA, CA, USA).

### Purification of prokaryotic-expressed Annexin A2

We transformed the *E*. *coli* expression strain, BL21(DE3), with pEXS-CG-Annexin A2 (GST-tagged construct), and cultured the cells in Luria-Bertani (LB) liquid medium with 50 μg/mL ampicillin at 37 °C until the density reached an OD_600_ of 1.5. Cells were cooled to 16 °C and cultured for another 12 h at 16 °C with 1.0 mM IPTG for protein expression. Then, cells were harvested by centrifuging at 6,000 rpm (JA10 rotor, Beckman) for 12 min. The cell pellet was resuspended in pre-cooled lysis buffer (PBS containing 1 mg/mL lysozyme, 1 mM DTT, and 1 mM PMSF), placed on ice, and ultra-sonicated for cell lysis. The lysed cells were separated with centrifugation at 12,000 rpm (JA25.50 rotor, Beckman) for 40 min, and the supernatant was loaded onto a GST-affinity chromatography column. The column was washed with pre-cooled PBS containing 1 M NaCl, 1 mM DTT, and 1 mM PMSF. Then, we added 10 μg of Human HRV 3 C Protease (TAKARA) to the column, and incubated the column at 4 °C overnight to allow cleavage of the GST-tag at the C-terminus of the protein. The eluted fraction was then loaded onto a Superdex75 16/600 column (GE Healthcare) and eluted at a flow rate of 1.0 mL/min. Fractions were pooled and concentrated to 10 mg/mL with a 10-kD cut-off centrifuge filter (Millipore).

The Annexin A2-K302A mutant was purified with the same procedure.

### Competitive (20S/R)G-Rh2-pulldown assay

*In vivo*: HepG2 cells were harvested and lysed with lysis buffer (Pierce) containing protease inhibitors (Roche). Aliquots of whole-cell lysate (2 mg) were diluted with lysis buffer to a final volume of 500 μL. Next, Rh2 was added to the diluted lysate for a final concentration of 3.75 μg/mL, and the same volume of 75% alcohol was added to a negative control preparation. The diluted lysate was placed in a rotating agitator at 4 °C for 4 h. Finally, 20 μL of the diluted lysate was collected as input. Next, 10 μL of (20S/R)G-Rh2-PEGA resin was subpackaged and washed three times with lysis buffer. The resin was resuspended in the diluted lysate and placed in a rotating agitator at 4 °C for another 4 h. The supernatant was removed, and the resin was washed three times with lysis buffer. The proteins were separated with SDS-PAGE, and immunoblot was performed to determine the presence of Annexin A2.

*In vitro*: 200 ng of purified Annexin A2 was diluted with lysis buffer in a final volume of 500 μL. The remaining steps were performed as described above for the *in vivo* competitive (20S/R)G-Rh2-pulldown assay.

### Isothermal Titration Calorimetry

We investigated the interactions between Annexin A2 and (20S)G-Rh2 with isothermal titration calorimetry (MicroCal iTC200, Malvern). We titrated 40 μL of purified Annexin A2 protein (200 μM) into the sample cell, which contained 200 μL (20S)G-Rh2 (20 mΜ) at room temperature. Data analysis was performed assuming a one-binding-site model.

### Luciferase reporter assay

HepG2 cells (3 × 10^5^) were plated into 6-well plates and transfected with pNF-кB-TA-luc and pRL-CMV. Then, HepG2 cells were treated with (20S)G-Rh2, PMA, and etoposide at the indicated concentrations for 12 h. The luciferase reporter assay was performed with the Dual-Luciferase® Reporter Assay System (Promega, Madison, WI, USA).

### Immunoprecipitation

*In vivo*: HepG2 cells (1.6 × 10^6^) were plated into 100φ plates and treated with (20S)G-Rh2, PMA, and etoposide for 12 h. Cells were lysed in lysis buffer (Pierce) containing protease inhibitors (Roche). Then, 2 mg of total protein lysates were mixed with 10 μL of anti-Annexin A2 or anti-NF-кB antibody, and incubated at 4 °C for 3 h on a tube rotator. Protein A/G beads (Millipore) were washed three times with lysis buffer, then incubated at 4 °C for 4 h with the lysate antibody complexes. The protein-agarose bead complexes were washed three times with lysis buffer. Samples were then separated with SDS-PAGE and analyzed with immunoblot.

*In vitro*: Cultures of the *E*. *coli* expression strain, BL21 (DE3), were transformed with pEXS-CG, pEXS-Annexin A2 (label-free), and pEXS-NF-кB. Cells were cultivated in LB liquid medium containing 50 μg/mL ampicillin at 37 °C, until the cell density reached an OD_600_ of 0.4. Then, cells were cultivated with 1 mM IPTG for another 12 h. Cells were lysed with ultra-sonication in lysis buffer (100 mM PBS, 125 mM NaCl) containing protease inhibitors and 1 mg/mL lysozyme. Next, 1 mg of the protein lysate containing Annexin A2 and another 1 mg of the protein lysate containing NF-кB were incubated at 4 °C overnight with 3.75 μg/mL (20S)G-Rh2. As a control, 1 mg of protein lysate that contained neither Annexin A2 nor NF-кB was incubated in parallel. The remaining steps were performed as described above for the *in vivo* assay.

### Immunofluorescence

Glass cover slips were placed into the wells of a 24-well plate, and 5 × 10^4^ HepG2 cells were seeded into each well. After a 16-h incubation, cells were treated with (20S)G-Rh2, etoposide, and PMA for 2 h. Next, cells were fixed with 4% paraformaldehyde and 4% sucrose at 4 °C for 20 min. Then, cells were washed twice with PBST (100 mM PBS with 0.5% Tween-20). The fixed cells were permeabilized with PBST containing 0.2% Triton X-100 at 4 °C for 20 min, then washed three times with PBST. Permeabilized cells were incubated with blocking buffer (PBST containing 3% donkey serum) at room temperature for 1 h, then incubated with a primary antibody (mouse anti-Annexin A2 or rabbit anti-NF-кB, each diluted 1:200 in PBST with 5% BSA) overnight at 4 °C. Cells were washed three times with PBST and incubated with a secondary antibody (Cy™3 affinipure donkey anti-mouse IgG or Alexa Fluor® 488 affinipure donkey anti-Rabbit IgG, each diluted 1:200 in PBST with 5% BSA) for 2 h at room temperature. After the cells were washed twice with PBST, they were mounted, stained with PBST and 0.1% DAPI (Sigma), and analyzed with a fluorescence microscope.

### Cellular thermal shift assay

Cells (3 × 10^7^) were seeded into a 100-mm culture plate with 7.5 μg/mL (20S)G-Rh2 at 37 °C for 1 h. Control cells were incubated with the same volume of ethanol. Cells were cultivated and counted, then resuspended in PBS to a final density of 2 × 10^7^/mL. Each sample was subpackaged into 12 PCR tubes, with 100 μL per PCR tube, and heated with a thermal gradient, from 40 °C to 73 °C, for 3 min. Cells were freeze-thawed twice with liquid nitrogen, and the supernatant was separated by centrifugation at 20,000 ×*g* for 20 min. The supernatant (20 μL) was loaded onto an SDS-PAGE gel, and the separated proteins were transferred to a membrane for immunoblot.

### Cell viability assay

HepG2 cells were transfected with pGPU6-GFP-Neo-Annexin A2-4 and pGPU6-GFP-Neo-NC. Then, cells were plated (5 × 10^3^ per well) onto 96-well plates and treated with (20S)G-Rh2, PMA, and etoposide, at the indicated concentrations for 48 h. Cell viability was determined with the MTT assay.

HepG2 cells were transfected with pcs4-Annexin A2-WT-myc and pcs4-Annexin A2-K302A-myc. Then, cells were plated (5 × 10^3^ per well) onto 96-well plates and treated with (20S)G-Rh2 at the indicated concentrations for 72 h. Cell viability was determined with the MTT assay.

### Plate clone formation assay

HepG2 cells were transfected with pcs4-Annexin A2-WT-myc and pcs4-Annexin A2-K302A-myc. Then cells were plated (1 × 10^3^ per well) onto 6-well plates and treated with (20S)G-Rh2 at the indicated concentrations for 1 week. Cells were washed twice with PBS and fixed with pre-cooled methanol for 10 min, followed by crystal violet staining for 5 min. Then cells were washed twice with PBS and photographed.

### Semi-quantitative PCR

Whole-cell RNA was isolated with TRIzol (Invitrogen). Then, 5-mg aliquots of whole-cell RNA were used for cDNA synthesis with the EasyScript Reverse Transcriptase kit (Transgen). Genes were amplified with PCR (primers shown in Table S1) with the following thermal cycler program: 1 cycle at 94 °C for 5 min, followed by 25 cycles of 94 °C for 30 s, 58 °C for 30 s, and 72 °C for 90 s, with a final extension at 72 °C for 10 min. PCR products were analyzed with agarose gel electrophoresis.

### Gene expression in non-tumor liver tissue and hepatocellular carcinoma tissue

We retrieved data on the expression levels of Annexin A2 and anti-apoptosis genes from the Oncomine™ database (http://www.oncomine.org). We searched the database with the following primary filters: ‘Differential Analysis’ = ‘Cancer vs. Normal Analysis’ and ‘Cancer Type’ = ‘Liver Cancer’. The relevant genes were scanned, one by one, in a Roessler Liver, which contained 220 samples of non-tumor tissue and 225 samples of tumor tissue. Graphs and statistical analyses were downloaded and presented here.

### Statistical analysis

Data are presented as the mean ± S.D. Statistical significance was calculated with the Student’s *t*-test.

## Electronic supplementary material

Supplementary Material
