# Supplementary material for: The identification of molecular target of (20S) ginsenoside Rh2 for its anti-cancer activity
Source: Sci Rep. 2017 Sep 29;7:12408. doi: 10.1038/s41598-017-12572-4 (PMC5622071; doi:10.1038/s41598-017-12572-4)
Supplement: Supplementary file 1 — Supplementary Material [file 41598_2017_12572_MOESM1_ESM.pdf]

The identification of molecular target of (20S) ginsenoside Rh2 for its anti-cancer activity

Yu-Shi Wang[1], Yingjia Lin[1], He Li[1], Yang Li[1], Zhiguang Song[2], and Ying-Hua Jin[1]*

[1]Key Laboratory for Molecular Enzymology and Engineering of the Ministry of Education, College of Life Science, Jilin University, Changchun, Jilin, China,130012

[2]College of Chemistry, Jilin University, Changchun 130012, China

*Correspondence to: Key Laboratory for Molecular Enzymology, Engineering of the Ministry of Education, Jilin University, 2699 Qianjin Street, Changchun 130012, China

E-mail address: yhjin@jlu.edu.cn

Tel: +86-431-85155221

Fax: +86-431-85155221

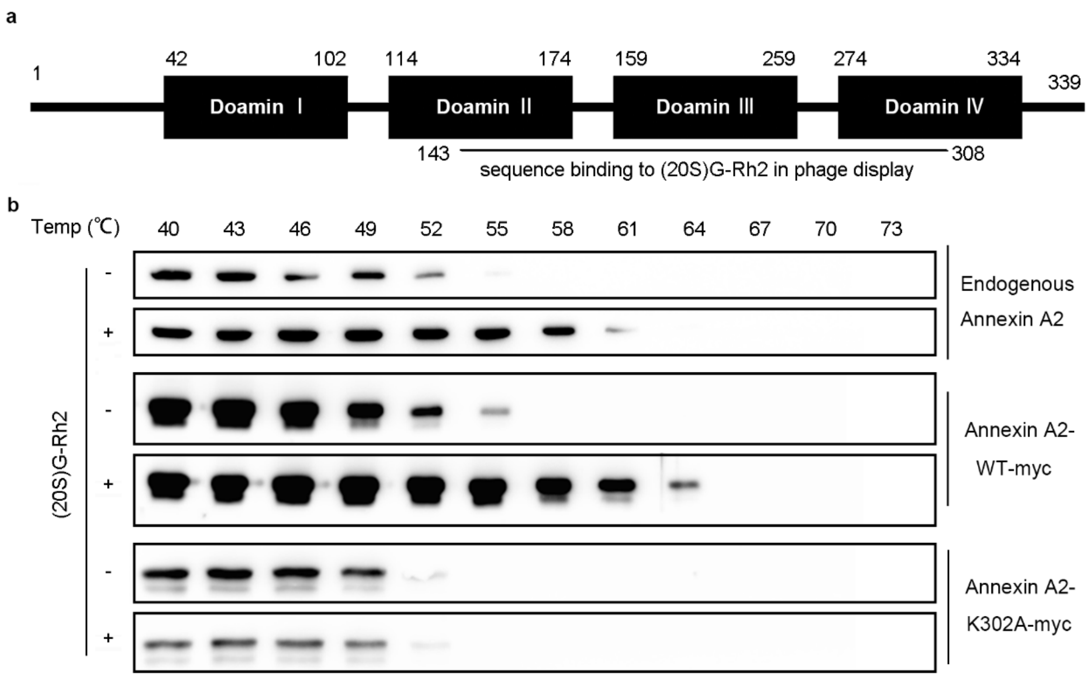

Figure S1 (20S)G-Rh2 binds to wild-type Annexin A2, but not the K302A mutant in vivo. (a) (*Top*) Numbers and line-block diagram indicate the structure of the full-length Annexin A2 protein sequence. (*bottom*) numbers and line indicate the Annexin A2 sequence identified here in the phage display. (b) Immunoblots of proteins from a cellular thermal shift assay show the effect of (20S)G-Rh2 on the temperature response of the Annexin A2-K302A or Annexin A2–WT expressed in HepG2 cells (endogenous) or HEK-293T cells (c-myc fusion proteins) .

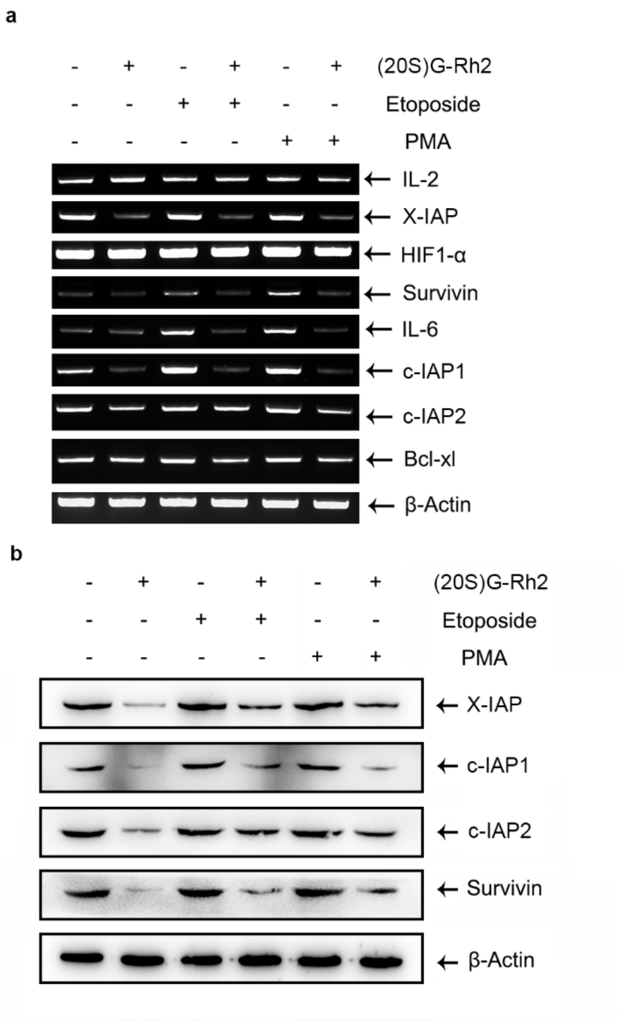

Figure S2 (20S)G-Rh2 down-regulated anti-apoptosis proteins and induced PARP cleavage. (a) Semi-quantitative PCR results show how (20S)G-Rh2 affects the mRNA levels of IL-2, X-IAP, HIF1-α, Survivin, IL-6, c-IAP1, c-IAP2, and Bcl-xL, in the absence (-) or presence (+) of 100 ng/mL PMA or 25 μg/mL etoposide. (b) Immunoblots show the levels of X-IAP, c-IAP1, c-IAP2, and Survivin proteins during treatment with 3.75 μg/mL (20S)G-Rh2, in the absence (-) or presence (+) of 100 ng/mL PMA or 25 μg/mL etoposide for 12 h.

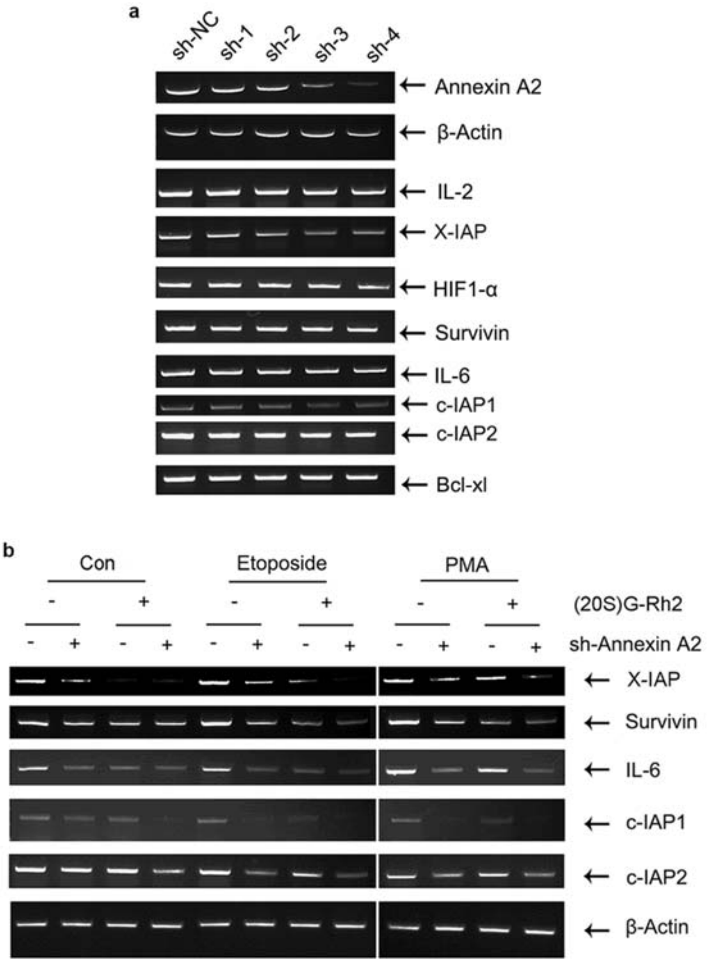

Figure S3 Downstream genes of NF-κB was down-regulated by both (20S)G-Rh2 and gene knockdown of Annexin A2. (a)HepG2 cells are transfected with 4 different shRNA targeting Annexin A2, semi- quantitative PCR results show mRNA levels of anti-apoptosis genes in HepG2 with different mRNA level of Annexin A2. (b) Semi-quantitative PCR results show mRNA levels of anti-apoptosis genes in HepG2 cells transfected with shRNA-Annexin A2 and treated with 3.75 μg/mL (20S)G-Rh2, 100 ng/mL PMA, and 25 μg/mL etoposide, as indicated. All data are shown as the mean ± SD and the experimental points show the average of at least triplicates. All experiments were repeated at least 3 times.

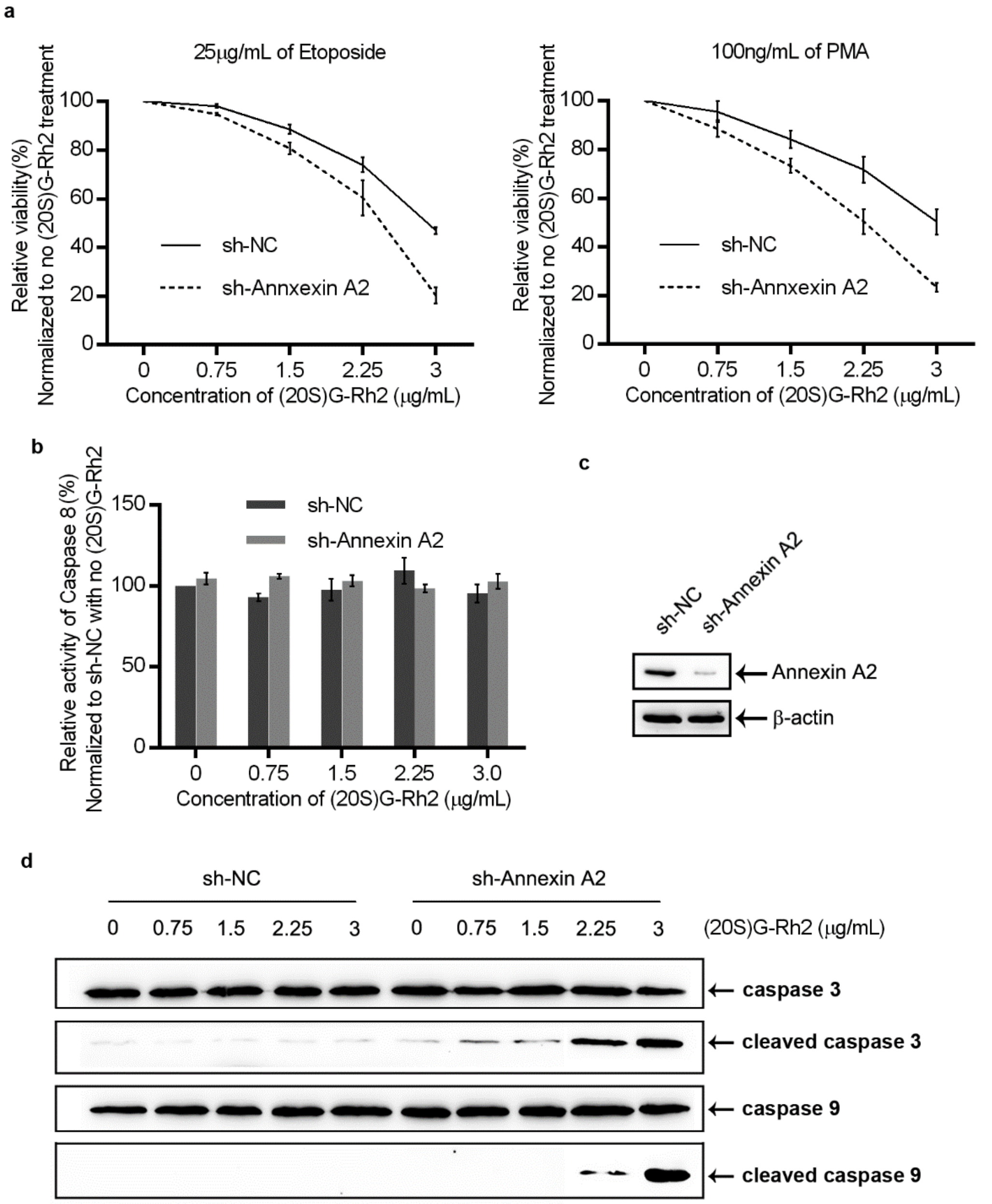

Figure S4 Knockdown of Annexin A2 enhanced apoptosis induced by (20S)G-Rh2.

(a) Cell viability in control (sh-NC) and Annexin A2-knockdown (sh-Annexin A2) HepG2 cells exposed to the indicated concentrations of (20S)G-Rh2, in the absence (Con) or presence of etoposide or PMA. (b) Relative activity of caspases 8 in HepG2 cells with normal Annexin A2 levels (sh-NC) or low Annexin A2 levels (sh-Annexin A2) exposed to low concentrations of (20S)-G-Rh2.(c) Protein level of Annexin A2 is shown via immunoblot after HepG2 cells are transfected sh-NC and sh-Annexin A2

respectively. (d) Immunoblots show protein levels of cleavage caspase 3 and caspase 9 in HepG2 cells and Annexin A2-knockdown HepG2 cells exposed to the indicated concentrations of (20S)G-Rh2. All data are shown as the mean ± SD and the experimental points show the average of at least triplicates. All experiments were repeated at least 3 times.

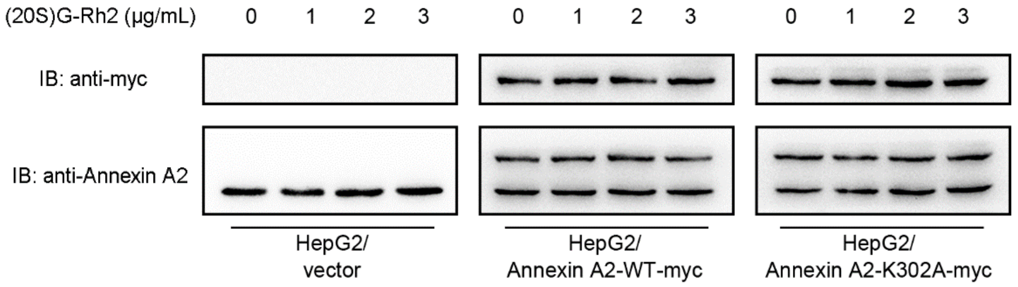

Figure S5 Over-expression of Annexin A2-WT-myc and Annexin A2-K302A-myc in HepG2 cells. Immunoblots show protein levels of Annexin A2 in HepG2 cells transfected with pcs4-Annexin A2-WT-myc and Annexin A2_K302A-myc, and exposed to (20S)G-Rh2 for 12 h. The levels of Annexin A2 protein were determined with anti-Annexin A2 and anti-myc antibodies.

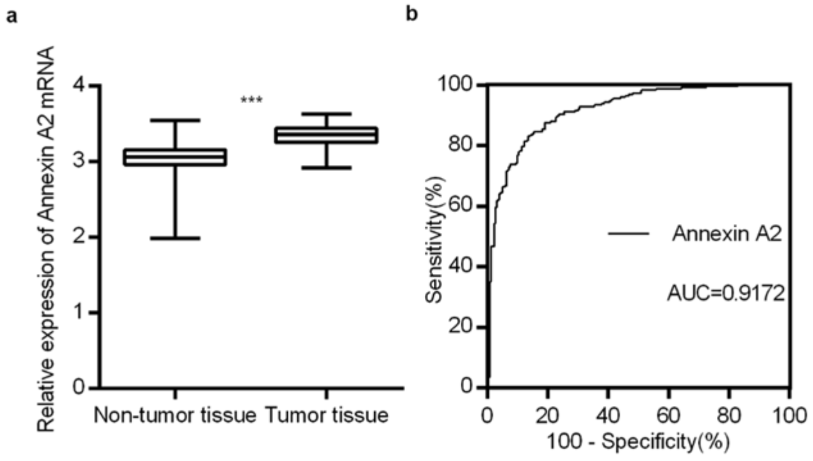

Figure S6: mRNA level of Annexin A2 was associated with tumor development. (a) Boxplots show mRNA levels of Annexin A2 in hepatocellular carcinoma (tumor tissue) and non-tumor tissues, based on data retrieved from the Oncomine database. All data are shown as the mean ± SD, ***P < 0.001. Statistical analyses were performed using Student's t-test. (b) Receiver operating characteristic curve of Annexin A2 in hepatocellular carcinoma, based on the relative expression of Annexin A2 in non-tumor and tumor tissues. the area under the curve (AUC) is 0.9172.

Table S1: Phage titers obtained after five rounds of bio-panning with (20R)G-Rh2-PEGA resin and the T7 Select Human Liver Tumor cDNA phage library

Round	Input phage	Elution phage	Elution (%)
Round 1	1×10^{11}	2.6×10^{4}	2.6×10^{-5}
Round 2	1×10^{11}	7.3×10^{4}	7.3×10^{-5}
Round 3	1×10^{11}	4.1×10^{5}	4.1×10^{-4}
Round 4	1×10^{11}	6.3×10^{5}	6.3×10^{-4}
Round 5	1×10^{11}	7.3×10^{5}	7.3×10^{-4}

Table S2: Primary analysis of genes obtained by sequencing phage plaques identified with the Rh2-PEGA resins

	(20S)G-Rh2-PEGA resin	(20R)G-Rh2-PEGA resin
Protein coding regions	95	138
Non-coding regions	47	60
No human genes	39	56
Total	181	254
Potential target proteins	46	65

Table S3: Primers for semi-qualitative PCR assay

Gene list	primers
IL2	5′-ACAGGATGCAACTCCTGTCT-3’<br>5′-GCACTTCCTCCAGAGGTTTGA-3’
IL6-	5′-CCAGTACCCCCAGGAGAAGA-3’<br>5′-CAGCTCTGGCTTGTTCCTCA-3’
X-IAP	5′-AGGCCATCTGAGACACATGC-3’<br>5′-TTCTGACCAGGCACGATCAC-3’
c-IAP1	5′-TCCAGCCTTTCTCCAAACCC-3’<br>5′-GCATGTGTCTGCATGCTCAG-3’
c-IAP2	5′-TGGAAGCTACCTCTCAGCCT-3’<br>5′-AAGCAAGCCACTCTGTCTCC-3’
Survivin	5′-GGACCACCGCATCTCTACAT-3’<br>5′-TGGCTCTTTCTCTGTCCAGT-3’
BCL2	5′-CATGTGTGTGGAGAGCGTCA-3’<br>5′-ACTTGTGGCCCAGATAGGCA-3’
BCLXL	5′-TCCCCATGGCAGCAGTAAAG-3’<br>5′-AGGTAAGTGGCCATCCAAGC-3’
HIF1A	5′-ACCTATGACCTGCTTGGTGC-3’<br>5′-TCCACCTCTTTTGGCAAGCA-3’

Table S4: Changes in the T_m50s of different Annexin A2 constructs with (20S)G-Rh2 treatment

	Control (℃)	7.5 µg/mL (20S)G-Rh2 (℃)	ΔT_m50 (℃)
Annexin A2_WT	47.50 ±0.05	57.20 ±0.80***	9.71 ±0.83
Annexin A2_WT-myc	49.48 ±0.11	58.44 ±0.22***	8.96 ±0.18
Annexin A2_K302A-myc	48.67 ±0.07	48.45 ±0.07	-0.23 ±0.11

Table S5: Changes in the expression of Annexin A2 and anti-apoptosis genes in hepatocellular carcinoma and non-tumor liver tissue.

Genes	Fold change(Cancer/Non-tumor)	P value
Annexin A2	3.506	1.10×10^{-65}
c-IAP1	1.142	9.20×10^{-6}
c-IAP2	1.835	3.23×10^{-17}
Survivin	3.441	5.83×10^{-67}
X-IAP	0.560	0.8714
